# Spinodal decomposition in alkali feldspar studied by atom probe tomography

**DOI:** 10.1007/s00269-020-01097-4

**Published:** 2020-06-07

**Authors:** Elena Petrishcheva, Lisa Tiede, Kevin Schweinar, Gerlinde Habler, Chen Li, Baptiste Gault, Rainer Abart

**Affiliations:** 1 Department of Lithospheric Research, University of Vienna, Althanstrasse 14, 1090 Vienna, Austria; 2 Max-Planck-Institut für Eisenforschung GmbH, Max-Planck-Straße 1, 40237 Düsseldorf, Germany; 3 Electron microscopy for Materials research (EMAT), University Antwerpen, Groenenborgerlaan 171, 2020 Antwerp, Belgium; 4 Department of Materials, Royal School of Mines, Imperial College, Prince Consort Road, London, SW7 2BP UK

**Keywords:** Alkali feldspar, Spinodal decomposition, Coherent solvus, Gradient energy, Atom probe tomography

## Abstract

We used atom probe tomography to complement electron microscopy for the investigation of spinodal decomposition in alkali feldspar. To this end, gem-quality alkali feldspar of intermediate composition with a mole fraction of $a_{\text {K}}=0.43$ of the K end-member was prepared from Madagascar orthoclase by ion-exchange with (NaK)Cl molten salt. During subsequent annealing at $550\,^\circ \hbox {C}$ and close to ambient pressure the ion-exchanged orthoclase unmixed producing a coherent lamellar intergrowth of Na-rich and K-rich lamellae. The chemical separation was completed, and equilibrium Na–K partitioning between the different lamellae was attained within four days, which was followed by microstructural coarsening. After annealing for 4 days, the wavelength of the lamellar microstructure was $\approx 17\,\hbox {nm}$ and it increased to $\approx 30\,\hbox {nm}$ after annealing for 16 days. The observed equilibrium compositions of the Na-rich and K-rich lamellae are in reasonable agreement with an earlier experimental determination of the coherent solvus. The excess energy associated with compositional gradients at the lamellar interfaces was quantified from the initial wavelength of the lamellar microstructure and the lamellar compositions as obtained from atom probe tomography using the Cahn–Hilliard theory. The capability of atom probe tomography to deliver quantitative chemical compositions at nm resolution opens new perspectives for studying the early stages of exsolution. In particular, it helps to shed light on the phase relations in nm scaled coherent intergrowth.

## Introduction

Alkali feldspar is a widespread phase in magmatic, metamorphic, and sedimentary rocks. It forms a solid-solution between a Na- ($\hbox {NaAlSi}_{3}\hbox {O}_{8}$) and K- ($\hbox {KAlS}_{3}\hbox {O}_{8}$) end-member. With changing pressure and temperature, alkali feldspar undergoes several ordering, displacive, and diffusive phase transformations (Kroll et al. 1986; Smith and Brown 1988; Parsons 1994; Ribbe 1983). The resulting intracrystalline microstructures reflect the conditions and mechanisms of magmatic and metamorphic crystallization and may potentially be used as petrogenetic indicators (Parsons et al. 2013, 2015; Parsons 1978; Parsons and Lee 2005, 2009; Fitz Gerald et al. 2006; Sanchez-Munoz et al. 2016).

At temperatures above about $600\,^\circ \hbox {C}$, alkali feldspar shows complete miscibility between the $\hbox {NaAlSi}_{3}\hbox {O}_{8}$ and $\hbox {KAlS}_{3}\hbox {O}_{8}$ end-members. Towards lower temperatures, a solvus opens, and when an alkali-feldspar with an intermediate composition is annealed within the spinodal region of the phase diagram, it exsolves spontaneously. Exsolved alkali feldspar is common in both magmatic and metamorphic rocks, and the resulting microstructure is referred to as perthite. Exsolution starts with the development of minute periodic fluctuations of the Na/K proportions with typical wavelengths in the nanometer range. With time, the fluctuations evolve into distinct Na-rich and K-rich domains which usually take the form of a lamellar intergrowth. Once formed, the lamellae coarsen and adapt their compositions to changing *P-T* conditions until they are gradually frozen in with decreasing temperature.

The microstructures and compositional patterns of perthites contain information on cooling rates and have been used for reconstructing the thermal histories of magmatic (Yund and Chapple 1980; Brown et al. 1983; Brown and Parsons 1984; Menna et al. 2008; Parsons and Gerald 2011) and metamorphic rocks (Day and Brown 1980; Evangelakakis et al. 1993; Abart et al. 2009b). The dynamics of exsolution by spinodal decomposition can be described by the Cahn–Hilliard theory (Cahn and Hilliard 1958), which was used for modelling spinodal decomposition of alkali feldspar and the successive coarsening (Abart et al. 2009b; Petrishcheva and Abart 2009, 2012). Whereas the Cahn-Hilliard theory yields excellent qualitative results, its potential for quantitative predictions is hampered by limited knowledge of the model parameters.

Although the thermodynamics of the alkali feldspar solid-solution is well established (Fuhrman and Lindsley 1988; Hovis 1988, 2017; Hovis et al. 1991; Benisek et al. 2010, 2014, 2015), uncertainties still exist with respect to the exact shape and position of the coherent solvus that is applicable to exsolution. Lamellar intergrowth generated by exsolution in alkali feldspar is usually coherent, and the associated strain energy counteracts the exsolution. As a consequence, the critical temperature for coherent exsolution is about 70 K lower than for incoherent intergrowth (Robin 1974; Sipling and Yund 1976; Tullis and Yund 1979; Brown and Parsons 1984). It must be noted that calculation of the elastic strain energy for constructing the coherent solvus from thermodynamic data, requires knowledge of the elastic constants (Robin 1974; Tullis and Yund 1979). Moreover, in the experimental determination of the coherent solvus by Sipling and Yund (1976) the compositions of the lamellae were determined indirectly from the lattice distortion in coherent intergrowth, a method that also relies on elastic constants (Yund and Tullis 1983). The elastic constants of alkali feldspar are, however, subject to considerable uncertainties (Waeselmann et al. 2016), and, especially for alkali feldspar of intermediate composition, they are poorly constrained. Hitherto, direct determination of lamellar compositions in coherent intergrowth was hampered by their small size. Atom probe tomography (APT) now offers possibilities for quantitative compositional analysis of nanometer sized microstructural features (Devaraj et al. 2018; Saxey et al. 2018; White et al. 2018). Here, we used APT for analyzing the average chemical compositions of the exsolution lamellae in nm-scaled coherent intergrowth, and in this way we could directly determine the position of the coherent solvus at $550\,^\circ \hbox {C}$ and close to ambient pressure. The spontaneous exsolution during spinodal decomposition is moderated by the free energy contribution of the newly formed transition zones between the Na-rich and the K-rich lamellae. We use the initial wavelength of the lamellar intergrowth and lamellar compositions as obtained from APT to quantify the free energy contribution of a diffuse coherent interface between two alkali feldspars of binodal compositions at $550\,^\circ \hbox {C}$ and close to ambient pressure.

## Experiment

*Starting material:* Gem-quality orthoclase from Madagascar was used as a starting material. A representative composition of Madagascar orthoclase obtained from electron probe micro analysis (EPMA) is given in Table 1. The Madagascar orthoclase is an essentially binary solid-solution between the $\hbox {NaAlSi}_3\hbox {O}_8$ and $\hbox {KAlSi}_3\hbox {O}_8$ feldspar end-members, whereby the mole fraction of the $\hbox {KAlSi}_3\hbox {O}_8$ end-member $a_{\text {K}}$ is 0.94. The orthoclase contains about 1.1 wt.% FeO, which very likely is present as tetrahedrally coordinated $\hbox {Fe}^{3+}$ (Hofmeister and Rossman 1984) and produces a yellow color. The space group is C2/m. The distribution of Al and Si on the T1 and T2 tetrahedral sites is charactericed by an intermediate topochemically monoclinic state of order. The Al content of the two T1 sites of a formula unit is $\Sigma _{\text {t1}} = 0.70$ (Neusser et al. 2012) compared to 0.77 as found by Hovis (1988). The extreme values of $\Sigma _{\text {t1}}$ range from 0.5 for full disorder to 1 for full order. The crystals are free of inclusions, cracks, or any other flaws.

*Cation exchange experiment:* The orthoclase was shifted to the composition $a_{\text {K}} \approx 0.43$ by cation exchange with an NaCl-KCl salt melt. To this end, the crystals were crushed and sieved to the size range of 63– $125\,\mu \hbox {m}$. The crystal fragments were loaded into quartz glass tubes with an inner diameter of 8 mm and 1.8 mm wall thickness that had been closed on one end before. Relying on the Na–K partitioning between alkali feldspar and salt melt at $850\,^\circ \hbox {C}$ as determined by Neusser et al. (2012), an NaCl–KCl salt mix with $a_{\text {KCl}} = 0.21$ was added to the feldspar to attain the target composition of $a_{\text {K}} \approx 0.43$. A 40 fold excess in terms of the alkali cations in the salt as compared to the alkali cations in the feldspar was applied to ensure essentially constant composition of the salt during the exchange experiment. The quartz glass tubes were then sealed under vacuum. The assembly was kept at $850\,^\circ \hbox {C}$ for 14 days to facilitate Na–K exchange. After the exchange experiment the glass vials were quenched in cold water, and the feldspars were retrieved by dissolving the salt with deionized water.

*Annealing experiment:* After cleaning and drying the exchanged feldspar fragments were loaded into quartz glass tubes again, now without the addition of salt, and the glass vials were sealed shut under vacuum. The samples were then annealed at $550\,^\circ \hbox {C}$ for 4, 8, and 16 days. After annealing the samples were quenched in cold water, and the powders were retrieved.

**Fig. 1:**
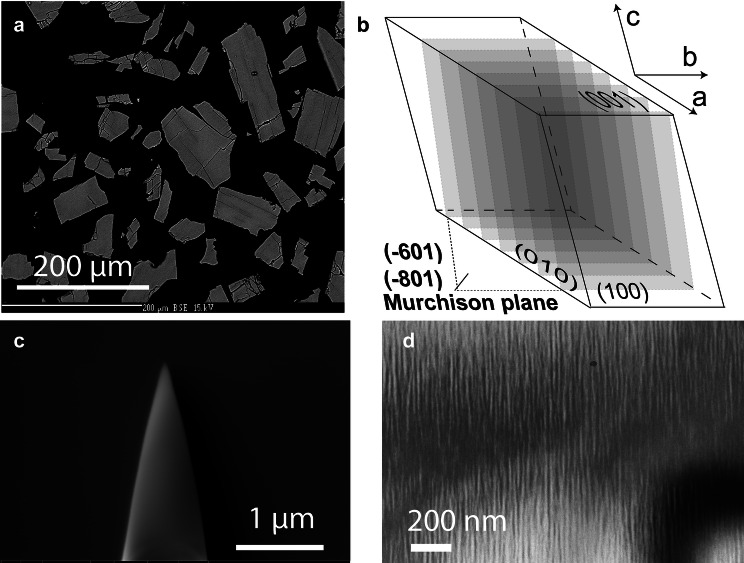
**a** BSE image of a polished grain mount of cation exchanged MO; **b** schematic drawing of a feldspar bounded by (100), (010) and (001) facets; the planes shown in transparent grey shades indicate the typical orientation of exsolution lamellae—Murchison plane with Miller indices in the range between ($\bar{6}01$) and ($\bar{8}01$); **c** APT tip prepared with Focused Ion Beam (FIB) technique; **d** TEM bright field image showing lamellar structure after annealing of the cation exchanged MO ex at $550\,^\circ \hbox {C}$ for 16 days

*Electron beam micro analysis:* Grain mounts (see Fig. 1a) were prepared from the run products and checked for homogeneity and composition using a CAMECA SX Five electron probe micro analyser (EPMA) at the Department of Lithospheric Research, University of Vienna. A representative EPMA analysis of the exchanged feldspar is given in Table 1. The crystal fragments have angular shapes indicating that they preferentially broke along the cleavage planes during crushing. Abundant cracks are visible that are aligned in a subparallel manner and run at high angles relative to both the (010) and the (001) cleavage planes. These cracks have been described earlier and are related to the anisotropic contraction of the crystal lattice during a compositional shift towards more Na-rich compositions (Neusser et al. 2012; Scheidl et al. 2014; Petrishcheva et al. 2019).

**Table 1:** Representative compositions, calculated cation numbers, and end-member mole fractions of Madagascar orthoclase: MO: original Madagascar orthoclase, MO ex: Madagascar orthoclase equilibrated with NaCl-KCl molten salt with $a_{\text {KCl}} = 0.21$ at $850\,^\circ \hbox {C}$ and 1 bar, a.p.f.u. indicates atoms per formula unit

	MO	MO ex
wt. % oxides		
$\hbox {SiO}_2$	66.18	66.34
$\hbox {Al}_2\hbox {O}_3$	17.25	18.04
FeO	1.06	1.13
CaO	0.00	0.00
$\hbox {Na}_2\hbox {O}$	0.54	6.40
$\hbox {K}_2\hbox {O}$	14.92	7.40
Total	99.95	99.43
a.p.f.u. based on 5 cations		
Si	3.08	3.00
Al	0.95	0.96
$\hbox {Fe}^{3+}$	0.00	0,02
$\hbox {Fe}^{2+}$	0.04	0,02
Ca	0.00	0.00
Na	0.05	0.56
K	0.83	0.43
End-member mole fractions		
$a_{\text {An}}$	0.00	0.00
$a_{\text {Ab}}$	0.06	0.57
$a_{\text {Or}}$	0.94	0.43

*Crystal orientation analysis:* The lateral resolution of chemical analysis by APT is highest in the direction parallel to the axis of the APT tip (see Fig. 1c). The APT tips were extracted with their long axis perpendicular to the sample surface. In order to attain the best possible lateral resolution in chemical analysis across lamella boundaries, grains with their exsolution lamellae oriented approximately parallel to the sample surface were selected for extracting APT tips. Exsolution lamellae in alkali feldspar are parallel to the Murchison plane, which has Miller indices in the range of $(\bar{6}01)$ to $(\bar{8}01)$ (Fig. 1b). The crystallographic orientations of the different crystal fragments were determined using electron backscatter diffraction (EBSD), and grains with the Murchison plane sub-parallel to the sample surface were selected for extraction of the APT tips. With this procedure, an orientation of the lamella approximately perpendicular to the length axis of the APT tip could be ensured, and the best possible lateral resolution in APT analysis could be obtained. The EBSD analyses were performed on an FEI Quanta 3D FEG-SEM at the laboratory for scanning electron microscopy and focused ion beam (FIB) applications of the Faculty of Geosciences, Geography and Astronomy at the University of Vienna (Austria).

*Scanning Transmission Electron Microscopy (STEM):* Scanning transmission electron microscopy (STEM) analyses were done for studying the nano-scale structure of the feldspar. Electron-transparent foils for STEM applications were prepared using the focused ion beam (FIB) technique on the same FEI Quanta 3D FEG-SEM instrument as used for EBSD analysis. A 30 kV Ga-ion beam was used for the pre-cut at high current (65–1 nA). An OmniprobeTM 100.7 micromanipulator was used for extracting the specimens. Then a 30 kV Ga-ion beam was used to thin the specimens to $\approx 50\hbox {nm}$ with lower current (1 nA–50 pA). Finally, a 5 kV and 2 kV Ga-ion beam was used for cleaning the specimens with very low current (48–27 pA).

**Fig. 2:**
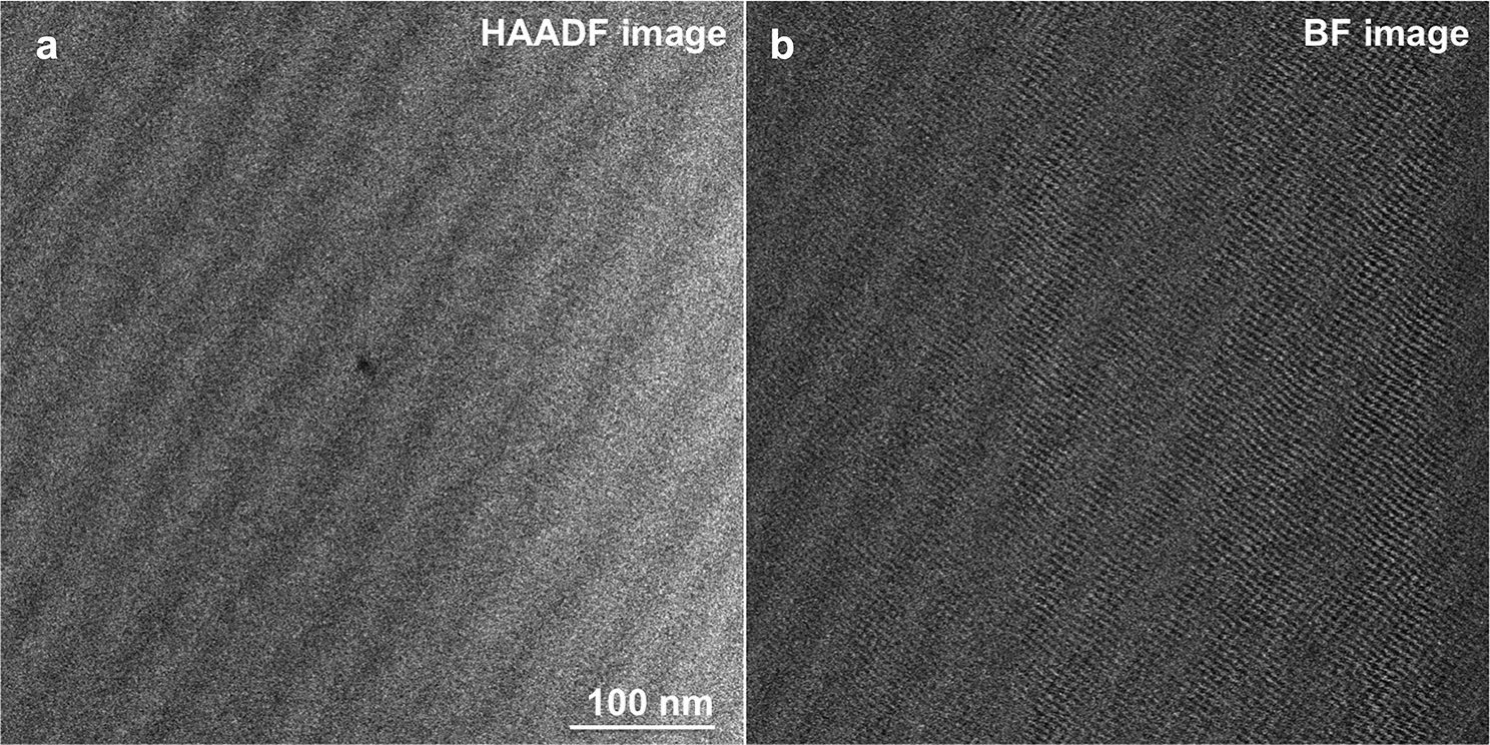
**a** STEM HAADF image with the viewing direction sub-parallel to a the [010] direction of the feldspar showing periodic intensity fluctuations reflecting variations in the Na- and K- concentrations in exsolved alkali feldspar from the 16 days annealing experiment. **b** STEM BF image of the same area as in image (**a**) showing fringes of a lattice plane crossing the lamellar interfaces coherently

A Nion UltraSTEM 5th-order aberration-corrected STEM was operated at 60 kV to resolve the crystal structure of the feldspar lamella. The probe-forming angle and the inner detector angle for the STEM high angle annular dark field (HAADF) images were approximately 30 and 80 mrad, respectively. A bandpass filter was used to remove the horizontal scanning noise from both the HAADF image and the BF image in Figure 2.

*Atom probe tomography:* Specimens for APT were prepared following the in-situ lift-out protocol detailed in Thompson et al. (2007), using a FEI dual beam Helios 600i focused-ion beam/scanning electron microscope (FIB/SEM) at MPI for Iron Research Düsseldorf. The specimens were placed on a silicon microtip coupon and sharpened by applying annular milling patterns at 30 kV acceleration voltage with FIB currents ranging from 0.23 nA to 46 pA. Low kV milling at 5 kV acceleration voltage and 40 pA was applied at the end to minimise Ga contamination at the surface of the samples. APT experiments were performed in a CAMECA LEAP 5000 XR instrument equipped with an ultraviolet laser with a spot size of approximately $2\, \mu \hbox {m}$ and a wavelength of 355 nm. The ion detection efficiency is reported to be 52 %. Data were acquired in laser pulsing mode at a base temperature of 50 K, with an average target detection rate of 0.01 atoms per laser pulse, a pulsing rate of 200 kHz or 250 kHz and a laser pulse energy of 400 pJ. The APT data were analysed with the commercial software IVAS 3.8.2.

**Fig. 3:**
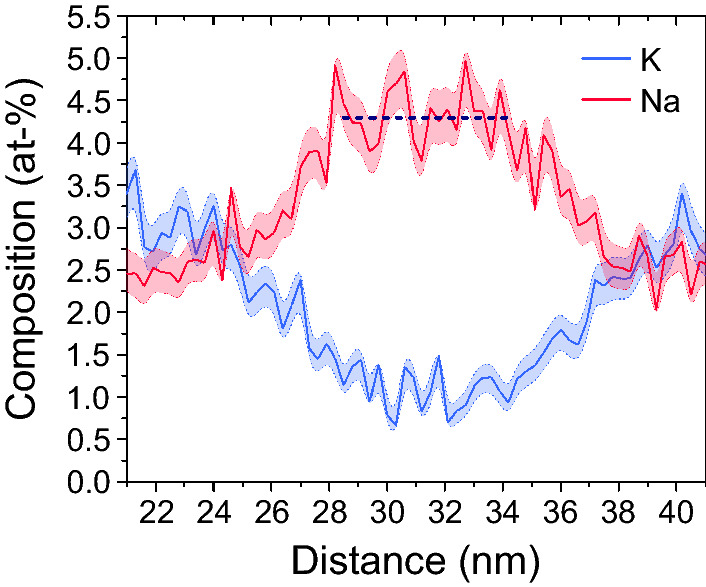
Representative Na- and K-profile across an about 13 nm wide Na-rich lamella as obtained from APT; horizontal line represents a fit to the plateau like part of the profile from which the average Na-concentration of the lamella was determined

A typical profile showing the variation of the Na- and K-concentrations (in at. %) across a Na-rich lamella is illustrated in Figure 3. The respective width of each of the lamellae was determined from the intersections of the Na- and K-composition curves. The average composition of an individual lamella was determined by fitting a straight line through the plateau-like sections of the composition profiles.

## Results

### Lamellar microstructure

STEM images of the lamellar microstructure of exsolved alkali feldspar from the 16 days annealing experiment viewed approximately parallel to the [010] direction of the feldspar are shown in Fig. 2. The intensity variations on the high-angle annular dark-field (HAADF) image approximately correspond to the squared ratio of the average atomic numbers of the compositionally distinct domains of the specimen. The HAADF image in Fig. 2a reveals a periodic intensity fluctuation, which is ascribed to the variation of the Na- and K- concentrations. The chemical segregation takes the form of lamellae with an apparent wavelength $\bar{\lambda }$ of 30 to 40 nm. Note that the TEM foil is oriented at an oblique angle relative to the lamellae so that $\bar{\lambda } \ge \lambda$, where $\lambda$ is the true wavelength defined as the average distance between the central planes of two neighboring lamellae of the same type. The relatively bright lamellae corresponding to the K-rich domains appear somewhat thicker than the relatively dark lamellae representing the Na-rich domains. The bright field (BF) image in Fig. 2b shows the moire patterns supposedly reflecting the traces of lattice planes. Due to the beam sensitivity of the sample, the electron dose on the sample needs to be kept low, which limits the resolution of the image. Nevertheless, it can be seen that the fringes of the lattice planes running approximately perpendicular to the lamellae are continuous across the lamellar interfaces. At any rate, the STEM images do not provide evidence for the presence of misfit dislocations at the lamellar interfaces testifying to the coherent nature of the lamellar intergrowth.

**Fig. 4:**
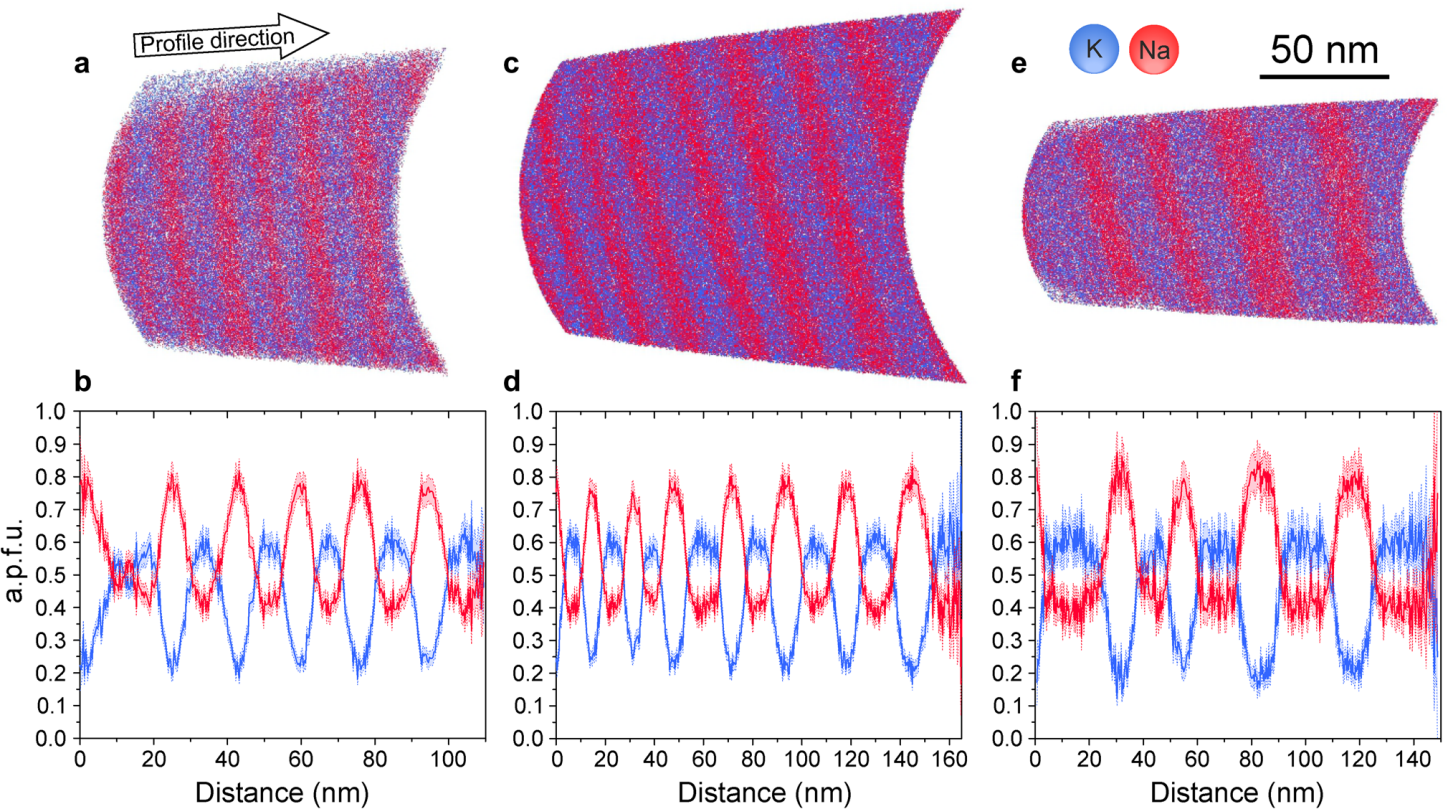
Reconstructed sections through APT tips extracted from the **a** 4 days, **c** 8 days, **e** 16 days experiment and the corresponding concentration profiles; **b**, **d**, **f** the sections are oriented in such a way that the Na-rich (red) and K-rich (blue) lamellae extend in viewing direction and, hence, show their true thickness, a.p.f.u. denotes atoms per formula unit

Sections through the specimens as reconstructed from the APT data and the extracted Na- and K compositional profiles obtained from the three tips prepared from the 4, 8, and 16 days annealing experiments are shown in Fig. 4. The reconstructions feature alternating Na-rich and K-rich lamellae. The viewing direction was chosen so that the lamellae are shown edge-on, and their true width is revealed. It is seen qualitatively that the K-rich lamellae (blue) are somewhat thicker than the Na-rich lamellae (red), which is in line with the STEM observations. The alternation of Na-rich and K-rich lamellae is quite regular, and a characteristic wavelength of the lamellar microstructure can be determined for each specimen. It is seen in Fig. 4 that the lamellar widths and the average wavelength of the lamellar microstructure tend to increase from the 4 days to the 16 days sample. Beyond this general trend, the lamellar widths show considerable variability within each specimen. In the section through the specimen from the 4 days sample, a bifurcation of a K-rich lamella is seen close to the left end of the reconstructed volume (Fig. 4a).

It must be noted that an accurate determination of lamellar widths from APT data requires an appropriate calibration of the tomographic reconstruction. When atomic planes can be imaged, this calibration is usually done by adjusting the reconstruction parameters so as to obtain an appropriate interplanar distance (Gault et al. 2009). In the absence of transmission electron imaging performed directly on the specimen (Herbig 2018; Liebscher et al. 2018), the tomographic reconstruction is usually constrained based on the shape or size of a specific microstructural feature (Larson et al. 2011). In the alkali feldspar, atomic planes could not be resolved, which is common for APT imaging of non-metallic materials. This is why the reconstruction parameters were optimised to ensure that the interfaces between the Na-rich and K-rich lamellae are flat, as observed with STEM in similar samples (Fig. 2). Even if the geometry of microstructural features is reproduced in a satisfactory manner, errors in the estimated width may still arise from an overestimation or an underestimation of the initial radius of the reconstruction $r_0$. We tested the influence of a change of $r_0$ by $\pm 10\%$ across the different data sets obtained here. Decreasing the $r_0$ yields physically meaningless curved interfaces. Increasing $r_0$ by $10\%$ results in a relative change of lamellar widths between 1 and 9%. This provides an estimate of the error associated with lamellar widths.

### Phase compositions

The Na- and K-concentrations in the different lamellae can be quantified from the data shown in Figure 4. The extracted compositions, wavelength, lamellar width and modal proportions of the Na-rich and K-rich lamellae are summarized in Table 2. In order to estimate the accuracy at which the Na/K proportions can be determined the analytical yield for the two components must be known. Species specific losses in APT have been reported in several cases Miller (1981). In particular, species loss has been observed for anions in covalently or ionically bonded materials, such as oxides, nitrides, carbides or hydrides. Species loss of anions is probably due to their complex field evaporation behavior (Gault et al. 2016; Zanuttini et al. 2017; Peng et al. 2019; Chang et al. 2019). Cations seem to be less strongly affected by species specific losses, probably because of their electronic affinity. Based on models for field evaporation, K and Na have ionization energies of about 5 eV. Accordingly, the critical electrostatic field necessary to provoke their field evaporation is nearly identical for both ionic species (Tsong 1978). This renders preferential loss of one of them rather unlikely, and the precision and the accuracy of the Na- and K-concentration measurement are very likely similar.

The extracted compositional profiles reveal a systematic pattern. The Na concentrations oscillate between about 0.4 a.p.f.u. in the K-rich lamellae and 0.8 a.p.f.u. in the Na-rich lamellae, and the K concentrations vary concomitantly from about 0.6 a.p.f.u. in the K-rich lamellae to 0.2 a.p.f.u. in the Na-rich lamellae. The sum of Na and K concentrations is thus $\approx 1$ a.p.f.u. throughout the sample, which is in line with the stoichiometric constraint on the alkali sublattice of alkali feldspar. The compositional profiles show plateaus in the K-rich lamellae, whereby the Na- and K-concentrations in the different K-rich lamellae are quite uniform within each sample and are similar within error limits for the 4, 8, and 16 days experiments (see Table 2). The compositions of the K-rich lamellae are thus interpreted as equilibrium compositions defining a binodal point at the conditions of the experiment. Plateaus cannot be discerned in most of the Na-rich lamellae, probably because they are too narrow. It is important to note that the compositions corresponding to the minima of the K-profiles and the maxima of the Na-profiles in the Na-rich lamellae do not change in any systematic manner with increasing run duration. They are thus also interpreted as equilibrium compositions corresponding to the second binodal point, accordingly.

The characteristic wavelength of the lamellar intergrowth increases from 17.5 nm after annealing for four days to 30.1 nm after annealing for 16 days. The standard deviation of the wavelength is substantially larger for the 8 and 16 days experiment than for the 4 days experiment indicating that prolonged coarsening leads to a higher variability of the lamellar widths. The modal proportions are approximately 45 vol. % Na-rich and 55 vol. % K-rich lamellae for all samples. The K-rich lamellae have an average composition of $a_{\text {K}} = 0.59$, the Na-rich lamellae have an average composition of $a_{\text {K}} = 0.24$ for the 4 days and 8 days sample and $a_{\text {K}} = 0.22$ for the 16 days sample. The integrated bulk composition of the lamellar intergrowth as calculated from combining lamellar compositions and modal proportions is $a_{\text {K}} = 0.42$ to $a_{\text {K}} = 0.44$, which perfectly matches the composition of the exchanged feldspar before the exsolution experiment ($a_{\text {K}} = 0.43$) as determined by electron probe micro analysis (see Table 1).

**Table 2:** Results from APT measurements; *t* is run duration in days; $\lambda$ and $\sigma$ are the average wavelength of the lamellar intergrowth and its standard deviation; the atoms per formula unit (a.p.f.u.) for Na and K refer to the mineral formula (Na,K)$\hbox {AlSi}_3\hbox {O}_8$; *n* indicates the number of lamellae used for determining lamella width and compositions; *m* is the modal (volume) proportions of the Na-rich and K-rich lamellae

bulk sample	M11		M12		M13	
*t* [d]	4		8		16	
$\lambda$($\sigma$) [nm]	17.5 (1.3)		21.2 (7.1)		30.1 (6.0)	
K [a.p.f.u.]	0.44		0.43		0.42	
Na [a.p.f.u.]	0.56		0.57		0.58	

lamellae	K-rich	Na-rich	K-rich	Na-rich	K-rich	Na-rich
*n*	5	5	7	7	5	4
width ($\sigma$) [nm]	10.6 (1.4)	8.3 (1.2)	12.0 (2.5)	9.7 (2.6)	17.0 (4.9)	14.9 (2.7)
*m*	0.56	0.44	0.55	0.45	0.53	0.47
K ($\sigma$) [a.p.f.u.]	0.59 ($<0.01$)	0.24 (0.01)	0.59 (0.01)	0.24 (0.02)	0.59 (0.01)	0.22 (0.03)
Na ($\sigma$) [a.p.f.u.]	0.41 ($<0.01$)	0.76 (0.01)	0.41 (0.01)	0.76 (0.02)	0.41 (0.01)	0.78 (0.03)

## Discussion

### Microstructural evolution

The spinodal decomposition of an initially homogeneous phase begins with the formation of minute compositional fluctuations, which may be described as spatial concentration variations with sinusoidal shape. Thereby, fluctuations with different wavelength are superimposed on one another. Increasing chemical segregation leads to the growth of the amplitudes of these fluctuations, where the rate of amplitude growth depends on wavelength. The fluctuations with the highest rate of amplitude growth dominate and define the characteristic initial wavelength of the chemical segregation. The chemical segregation proceeds until the binodal compositions, that are the compositions at which the segregated phases are in equilibrium with each other, are reached. The further evolution is dominated by microstructural coarsening. In lamellar intergrowth, such as is typical for exsolved alkali feldspar, coarsening occurs by the breakup of lamellae and their successive elimination (Yund 1984; Abart et al. 2009a, b). The breakup of a lamella necessarily produces a domain, where the two adjoining lamellae are merged into one, and two bifurcations flanking this domain. At the end-point of a disconnected lamella the curvature of the lamellar interface is much higher than along the continuous portions of the lamella driving retreat of the disconnected lamella (Brady 1987). Lamellar end-points and the associated bifurcations are thus the active sites of coarsening. In the section through the specimen from the 4 days experiment, a bifurcation of a K-rich lamella is seen close to the left end of the reconstructed volume (Fig. 4a). This indicates that the sample already had the potential for effective coarsening by the elimination of lamellae after annealing at $550\,^\circ \hbox {C}$ for four days. Such bifurcations very likely also exist in the specimens from the 8 and 16 days experiments, but they are not observed in Figures 4c, e probably because of the limited volume sampled by APT.

### Chemical equilibrium in coherent intergrowth

It has been shown by Abart et al. (2009b) that in the course of spinodal decomposition during cooling, the microstructural coarsening stops at higher temperature than chemical re-equilibration between the different lamellae by inter-lamellar Na–K exchange. This indicates, that the kinetics of lamellar growth is substantially more sluggish than the kinetics of compositional change by cation exchange. Thus, the fact that in our experiments the lamellar compositions did not change from the 4 to the 16 days annealing experiment while the characteristic width of the lamellae almost doubled during that period (Table 2) strongly corroborates the view that the Na–K partitioning between the lamellae indeed had reached equilibrium and that the observed lamellar compositions represent the binodal compositions of coherent two feldspar intergrowth at the conditions of the experiment.

The free energy of a binary alkali feldspar solid-solution may be expressed as

1 $$\begin{aligned} g(a)= g_{\text {K}}^0 a +g_{\text {Na}}^0(1-a) +RT\biggr [a \ln a +(1-a )\ln (1-a)\biggr ]+ g^\text {ex} , \end{aligned}$$

where *a* is the mole fraction of the K end-member component (we omit the subscript K in the equations for better readebility), and $g_{\text {Na}}^0, g_{\text {K}}^0$ are the Gibbs energies of the pure Na and K end-member phase components. The last two terms represent the configurational free energy and the excess Gibbs energy due to the non-ideality of the solid solution, respectively. For the excess Gibbs energy we use an asymmetric sub-regular solution model,

2 $$\begin{aligned} g^\text {ex}(a,P,T)= \left[ a\,W_{G,\text {KKNa}}(P,T) + (1-a)W_{G,\text {NaNaK}}(P,T) \right] a \left( 1-a \right) , \end{aligned}$$

where $W_{G,\text {KKNa}}$ and $W_{G,\text {NaNaK}}$ are the respective Margules parameters for the excess Gibbs energy, and

$$\begin{aligned} W_G = W_H - TW_S, \end{aligned}$$

with $W_H$ and $W_S$ being the Margules parameters for excess enthalpy, and excess entropy of mixing, respectively. We employ the Margules parameters derived from solid solutions prepared by ion-exchange from two different feldspars, analbite-sanidine and adularia (Hovis 2017) (see Table 3). Despite the fact that both feldspars are topochemically monoclinic, the solid-solution series prepared from analbite-sanidine has a substantially higher degree of Al–Si disorder in the tetrahedral framework than the solid-solution series prepared from adularia. There are no Marugles parameters available for Na–K exchange in a solid-soluiton series prepared from Madagaskar orthoclase, but direct calorimetric determination of excess enthalpies (Hovis 1988) inidcate that the non-ideality of Na–K mixing in orthoclase should be bracketed by the respective properties of the analbite–sanidine and the adularia series. The solvus for an orthoclase derived solid-solution series should thus lie between the solvus curves calculated for the analbite-sanidine and the adularia series.

**Table 3:** Margules parameters for excess enthalpy and excess entropy of Na–K mixing in topochemically monoclinic alkali feldspar; analbite–sanidine and adularia models are taken from Hovis (2017), the best fit model is modified from Hovis et al. (1991), their analbite–sanidine model with $W_{H,\text {KKNa}}$ adjusted to fit the binodal points observed in our experiments

model	$W_{H,\text {KKNa}}$	$W_{H,\text {NaNaK}}$	$W_{S,\text {KKNa}}$	$W_{S,\text {NaNaK}}$
	[$\hbox {J mol}^{-1}$]	[$\hbox {J mol}^{-1}$]	[$\hbox {J K}^{-1}\hbox { mol}^{-1}$]	[$\hbox {J K}^{-1}\hbox { mol}^{-1}$]
analbite-sanidine	17400	20630	10.99	-0.24
adularia	11940	44700	2.88	26.22
best fit	22000	22820	10.50	6.30

The lamellar intergrowth resulting from the spinodal decomposition in alkali feldspar is coherent, and the associated strain energy must be accounted for in the expression for the free energy. The lattice parameters of alkali feldspar strongly depend on composition (Kroll and Ribbe 1983). Generally, the lattice parameters of alkali feldspar shrink with increasing Na-content, whereby the effect is strongest in the direction sub-parallel to the crystallographic ***a*** and less pronounced in the crystallographic ***b*** and ***c*** directions. Starting from an initially homogeneous crystal, the crystal structure expands in the more K-rich domains emerging during exsolution, and it contracts in the more Na-rich domains. In coherent lamellar intergrowth, the Na- and K-rich domains are, however, mechanically coupled across the lamellar interfaces. For coherency to be maintained across the interface between a Na-rich and a K-rich domain, the lattices on either side of the interface must be elastically strained to compensate for the chemically induced lattice misfit. Typically, the interfaces orient themselves perpendicular to the direction with the largest chemically induced eigenstrain, so that the elastic strain energy associated with a lamellar alternation of Na-rich and K-rich domains is minimized. In alkali felspar, the interfaces between the Na-rich and the K-rich lamellae uniformly assume an orientation approximately parallel to the Murchison plane, which has an orientation of ($\bar{8}01$)–($\bar{6}01$), that is sub-perpendicular to the direction with the largest chemically induced eigenstrain. Coherency of the crystal lattice across the lamellar interfaces requires that the lattice of the Na-rich domains is expanded, and the lattice of the K-rich domains is contracted parallel to the interface plane. In the direction perpendicular to the lamellar interfaces, this leads to an elastic distension of the *d*-spacing in the K-rich lamellae and to an elastic contraction in the Na-rich lamellae. Thus, the lattices of both, the Na-rich and the K-rich lamellae are distorted relative to the lattice of a mechanically unconstrained feldspar of similar composition. The strain energy associated with coherent intergrowth counteracts exsolution and suppresses the coherent solvus relative to the strain free solvus. Robin (1974) and Tullis and Yund (1979) adapted Cahn’s theory on coherent intergrowth (Cahn 1962) for alkali feldspar and provided an expression for the free energy in coherent lamellar intergrowth

3 $$\begin{aligned} \phi = g(a) + k \left( a - a_0 \right) ^2 \end{aligned}$$

where *g*(*a*) is the compositionally-dependent Gibbs energy of a strain free alkali feldspar (Eq. 1), *a* is the composition of a lamella, $a_0$ is the bulk composition of the lamellar intergrowth, and *k* is the strain energy coefficient, a constant calculated from the compositional dependence of the lattice parameters combined with the elastic constants of alkali feldspar.

**Fig. 5:**
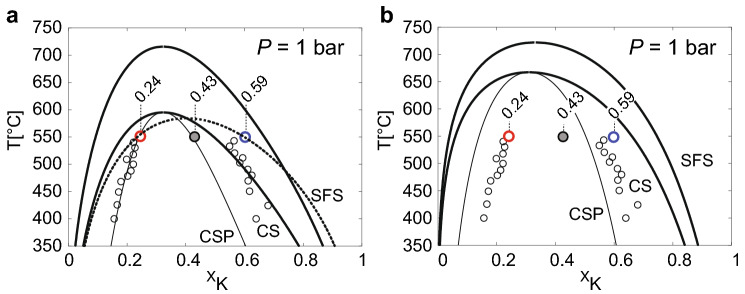
Alkali feldspar solvus calulated from the analbite–sanidine model (a) and from the adularia model (b) of Hovis (2017): SFS: strain free solvus; CS: coherent solvus and CSP coherent spinodal obtained from employing the strain energy after Robin (1974) ($k =2527\,\hbox {Jmol}^{-1}$); the small open circles are data from the exsolution experiments of Sipling and Yund (1976); the large heavy red and blue circles are the compositions of the Na-rich and the K-rich lamellae, respectively, as determined by APT; the large grey circle is the integrated bulk composition as determined from EPMA; the dashed heavy line in (a) is calculated from the best fit model (Table 3) modified from Hovis et al. (1991)

The strain free solvus derived from application of Gibbs energy minimization to the above analbite-sanidine and adularia models are shown as the outermost heavy solid lines labelled SFS in Fig. 5a, b. The corresponding solvus and spinodal curves for coherent intergrowth are shown as heavy and thin solid lines labelled CS and CSP, repsecrtively, whereby the strain energy coefficient was set to $k =2527\hbox { Jmol}^{-1}$ (Robin 1974).

In Figure 5, the original composition of the ion-exchanged homogeneous orthoclase ($X_{\text {K}}=0.43$) as determined from EPMA and of the Na-rich ($X_{\text {K}}=0.24$) and K-rich ($X_{\text {K}}=0.59$) lamellae as determined by APT are shown as grey, red, and blue circles, respectively. In addition, the data of Sipling and Yund (1976), who determined the coherent solvus of high sanidine by means of exsolution experiments, are shown as light black circles. The APT results for the Na-rich lamellae are in quite good agreement with the corresponding data of Sipling and Yund (1976). The composition of the K-rich lamellae as determined from APT is somewhat more K-rich than was determined by Sipling and Yund (1976). A more pronounced chemical segregation during exsolution of ion exchanged Madagaskar orthoclase as compared to exsolved high-sanidine is not surprising, because the distribution of Al-Si on the tetrahedral sublattice is more disordered in high-sanidine than in orthoclase, and increasing disorder of Al-Si decreases the excess Gibbs energy of mixing and thus the miscibllity gap (Hovis 2017). It is important to note, that the compositions given by Sipling and Yund (1976) were calculated from the lattice parameters determined by XRD combined with elastic constants. The elastic constants of alkali feldspar are, however, subject to considerable uncertainties (Waeselmann et al. 2016), and the calculated lamella compositions need to be considered with caution. In contrast, the compositions obtained by APT are perfectly in line with electron probe microanalyses of the bulk lamellar intergrowth and coincide for three independent experiments, indicating that they are reliable. In light of the fundamentally different analytical approaches and the inherent uncertainties, the direct determination of the coherent solvus by Sipling and Yund (1976) and our results are in remarkably good agreement.

The agreement between theoretical predictions and experimental determinations of the coherent solvus is less satisfactory. The curves labelled SFS, CS and CSP in Figure 5a represent the strain free solvus, the coherent solvus and the coherent spinodal calculated from the analbite–sanidine model of Hovis (2017). In Fig. 5b, the corresponding curves are shown for the adularia model of Hovis (2017). For both models the Na-rich limb of the solvus is predicted to be substantially more Na-rich than what has been determined experimentally. On the K-rich side of the solvus, the analbite-sanidine model predicts less chemical segregation then experimentally observed at $550\,^\circ \hbox {C}$, and the adularia model predicts more pronounced chemical segregation. This picture is compatible with the expected increase in excess Gibbs energy from analbite-sanidine via orthoclase to adularia. For the analbite-sanidine model the composition of the homogeneous precursor phase ($X_{\text {K}}=0.43$) is outside the spinodal curve, which is incampatible withe the experimentally observed, rapid and pervasive exoslution during annealing at $550\,^\circ \hbox {C}$, which testifies to a spinodal process.

It must be noted, that the determination of the Margules parameters is rather delicate. The parameters used for calculating the solvus and spinodal curves in Fig. 5 were obtained by Hovis (2017) by combining calorimetric measurements of excess enthalpies of Na–K mixing by solution calorimetry at $50\,^\circ \hbox {C}$ (Hovis 1988) and experimentally determined Na–K partitioning between alkali feldspar and (NaK)Br molten salt at $800\,^\circ \hbox {C}$. Thereby the specific values of the Margules parameters for the excess entropy are highly sensitive to the choice of data (compositional range considered) and the calculated solvus curves are subject to considerable uncertainty.

An additional source of uncertainty is related to the strain energy coefficient. The curves for coherent intergrowth were calculated using the strain energy coefficient of Robin (1974) ($k =2527\, \hbox {Jmol}^{-1}$). A somewhat smaller strain energy coefficient is obtained from the calculation after Tullis and Yund (1979) ($k =1925\,\hbox {Jmol}^{-1}$), respectively, when “soft” elastic constants and the $520\,^\circ \hbox {C}$ cell parameters are applied. Due to the uncertainty associated with the elastic constants of alkali feldspar (Waeselmann et al. 2016) the calculated strain energy coefficients need to be considered with caution.

The observed lamella compositions can be reproduced, when the strain energy coefficient is reduced to $k =1690\,\hbox {Jmol}^{-1}$ and $W_{\text {KKNa}}$ is changed from $1.1 \times 10^4\,\hbox {Jmol}^{-1}$ as is obtained from the model parameters of Hovis et al. (1991) to $1.3 \times 10^4\,\hbox {Jmol}^{-1}$ (best fit model in Table 3). It is important to note, that these parameters are optimized for the ordering state of Madagascar orthoclase with $\sum t_1 = 0.70$, which does not change during cation exchange at $850\,^\circ \hbox {C}$ and annealing at $550\,^\circ \hbox {C}$ (Neusser et al. 2012). The corresponding coherent solvus is shown as heavy dashed line Fig. 5a. It must be noted, that due to the lack of systematics in the differences between experimental calibrations of the coherent solvus and theoretical predictions this choice of parameters is highly arbitrary and should not be used as thermodynamic model for orthoclase-based solid solutions of alkali felspar.

### Initial lamellar width and gradient energy

Spinodal decomposition occurs by the spontaneous growth of compositional fluctuations with different wavelength, the amplitudes of which increase until the binodal compositions are reached. It can be shown in the frame of Cahn–Hilliard theory (Cahn and Hilliard 1958) that the characteristic wavelength of the initial chemical segregation depends on the gradient energy, that is the free energy associated with the compositional gradients that exist at the transitions between compositionally distinct domains. Based on the rationale that the characteristic initial wavelength of chemical segregation $\lambda _{\text {opt}}$ is the one with the fastest growth rate of its amplitude, a relation between the gradient energy parameter $\kappa$ and the characteristic wavelength can be derived (see Appendix)

4 $$\begin{aligned} \kappa = - \frac{\lambda _{\text {opt}}^2}{8 \pi ^2} \frac{\partial ^2 g}{\partial a^2}\bigg |_{a_0} , \end{aligned}$$

where $\partial ^2 g/\partial a^2$ is obtained from

5 $$\begin{aligned} \frac{\partial ^2 g}{\partial a^2}\bigg |_{a_0} = \frac{RT}{a (1-a)} + W_{\text {KKNa}} \left( 2 - 6a \right) + W_{\text {NaNaK}} \left( 6a - 4 \right) , \end{aligned}$$

which is taken at $a_0$, the composition of the homogeneous precursor phase, and $g = g(a)$ is taken from Eqs. 1–2.

We employ the best fit mixing model modified from Hovis et al. (1991) (Table 3), which yields $\partial ^2 g / \partial a^2 = -4.9 \times 10^{3}\hbox {J/mol}$ for a bulk composition of $a_0 = 0.43$ and an annealing temperature of $550\,^\circ \hbox {C}$ at 1 bar pressure. Based on the observation of successive coarsening, we take the average lamellar spacing observed after 4 days annealing at $550\,^\circ \hbox {C}$, which is $1.75 \times 10^{-8}\hbox {m}$, as an upper bound and a linear extrapolation of the thickness data (Table 2) to zero time, which yields $10^{-8}\hbox {m}$, as the lower bound for the feasible range of the initial lamellar spacing. Thus setting $\lambda _{\text {opt}} = 10^{-8}\hbox {m}$ to $1.75 \times 10^{-8}\hbox {m}$ we obtain a gradient energy parameter of $\kappa = 6.2 \times 10^{-15}\hbox {Jm}^2$ to $1.9 \times 10^{-14}\hbox {Jm}^2$. Inserting these values into Eq. (A-10) (Appendix) yields a minimum size of 7.1 nm to 12.4 nm, below which compositional fluctuations will not grow spontaneously, and spinodal decomposition cannot occur.

The excess free energy per unit area associated with the compositional gradient across a diffuse boundary between two alkali feldspars of binodal compositions is obtained from (Petrishcheva and Abart 2009)

6 $$\begin{aligned} \gamma =\nu \sqrt{\frac{\kappa }{2}} \int _{a_1}^{a_2} \sqrt{ g(a) - \frac{a_2-a_0}{a_2-a_1}g(a_1) - \frac{a_0-a_1}{a_2-a_1}g(a_2) }~da, \end{aligned}$$

where $\nu$ is the number of cation sites per unit volume in $\hbox {mol/m}^3, a_0, a_1$, and $a_2$ are the bulk composition and the binodal compositions of the feldspar, respectively, and $\kappa$ is taken from Eq. 4. Using the values $\kappa = 6.2 \times 10^{-15}\hbox {Jm}^2$ and $\kappa = 1.9 \times 10^{-14}\,\hbox {Jm}^2$ and the best fit mixing model (Table 3), which best reproduces the observed lamellar compositions, we obtain $\gamma = 10^{-3}\,\hbox {J/m}^2$ to $1.7 \times 10^{-3}\,\hbox {J/m}^2$. In comparison, the excess energy of a general alkali feldspar surface with an orientation close to the Murchison plane is about 0.9 to $1.1\,\hbox {J/m}^2$ as determined by Petrishcheva et al. (2019). The excess energy arising from the composition gradients that exist across a diffuse interface between two alkali feldspars with binodal compositions is thus by a factor of about 500–1100 smaller than the typical surface energy of alkali feldspar. This gradient energy is the driving force underlying coarsening in coherent intergrowth in alkali feldspar. The comparatively small excess energy associated with compositional gradients may serve as a rationale for the finding that coarsening driven by the gradient energy is much less efficient than an increase in grain size due to the growth of precipitates from a supersaturated feldspar solid solution, where the thermodynamic driving force is substantially higher (Abart et al. 2009a).

## Conclusions

The spinodal decomposition of alkali feldspar was investigated experimentally. To this end, an initially homogeneous, gem quality alkali feldspar with $a_{\text {K}} = 0.43$ prepared from Madagaskar orthoclase by cation exchange was annealed at $550\,^\circ \hbox {C}$ and close to ambient pressure. Within four days, a lamellar intergrowth of more Na-rich and more K-rich domains was produced. After four days the characteristic wavelength of the lamellar intergrowth was about 17 nm. During prolonged annealing for 8 and 16 days the lamella compositions remained unchanged, while the characteristic wavelength increased to about 30 nm after 16 days due to coarsening. Despite of the small size of the exsolution lamellae, their compositions could be quantified using atom probe tomography. A perfect match between bulk chemical analysis by electron probe micro analysis and the bulk composition calculated from lamella compositions and modal proportions as obtained from APT analysis was achieved testifying to the high accuracy that can be attained with APT analysis of nm-scaled lamellar intergrowth. The lamellar compositions of $a_{\text {K}} = 0.24$ and $a_{\text {K}} = 0.59$ are interpreted as binodal points pertaining to the Na-rich and K-rich limbs of the coherent solvus at $550\,^\circ \hbox {C}$ and ambient pressure. The binodal points are in good agreement with earlier experimental determinations of the coherent solvus in disordered alkali feldspar. They are, however, not well reproduced by thermodynamic models. The discrepancy between experimentally determined and theoretically predicted binodal compositions is ascribed to considerable uncertainties in the underlying thermodynamic mixing models and the strain energy of coherent intergrowth. The initial wavelength of chemical segregation and the equilibrium compositions of the lamellae as determined by APT were used for quantifying the excess free energy associated with the compositional gradient that exists across a coherent diffuse interface between two alkali feldspars of binodal compositions. At $550\,^\circ \hbox {C}$ and 1 bar, the excess energy is $1 \times 10^{-3}\,\hbox {J/m}^2$ to $1.7 \times 10^{-3}\,\hbox {J/m}^2$, which is smaller by a factor of about 500–1100 than the typical surface energy of a general plane in alkali felspar. The low excess energy associated with compositional gradients in alkali feldspar explains the low efficiency of coarsening in perthites produced from spinodal decomposition, where the gradient energy is the only thermodynamic driving force for coarsening. The capability of atom probe tomography for quantitative chemical analysis with nm-resolution provides a new avenue for determining phase relations in nano-structured materials. In particular, it allows for monitoring the incipient stages of exsolution and precipitation at unprecedented resolution and accuracy.

## Figures and Tables

**Fig. 6:**
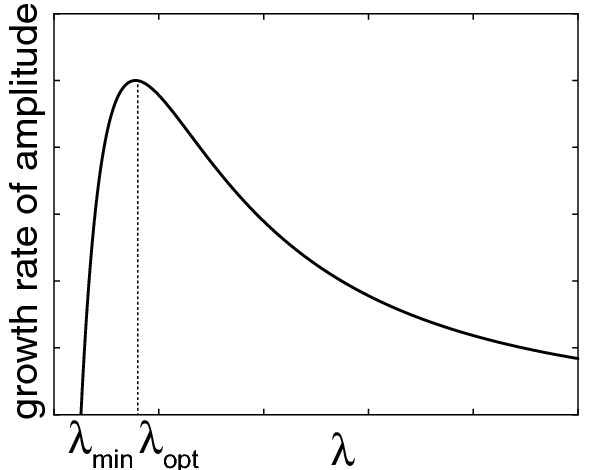
Schematic diagram showing the growth rate of the amplitude of a compositional fluctuation as a function of wavelength of the fluctuation. Spinodal decomposition is only possible for $\lambda > \lambda _{\text {min}}$, the amplitude growth rate has its maximum at $\lambda = \lambda _{\text {opt}}$

## Appendix

For a binary solution phase, the Cahn–Hilliard equation (CHE) reads (Cahn and Hilliard 1958)

A-1 $$\begin{aligned} \frac{\partial a}{\partial t} =\nabla \left[ \frac{D}{RT} a(1-a)\nabla \left( \frac{\partial g}{\partial a}- \kappa \nabla ^2a \right) \right] , \end{aligned}$$

where $a=a(\mathbf{r},t)$ and $(1-a)$ are the mole fractions of the two components, *D* is the interdiffusion coefficient, *R* is the gas constant, and *T* is absolute temperature. The free energy of a uniform mixture is denoted as *g*(*a*). The term $\kappa \nabla ^2a$ accounts for the free energy contribution of the compositional gradients that are associated with the phase boundaries between two compositionally distinct, immiscible members of the solution phase, $\kappa$ is the gradient energy parameter.

The compositional fluctuations developing during the incipient stages of spinodal decomposition may be described by a Fourier expansion, i.e., by a linear combination of sinusoidal functions of different wavelength. For simplicity we consider a small perturbation with only one harmonics along the *OX* axis

A-2 $$\begin{aligned} a = a_0 + \tilde{a}(t) \cos Kx, \qquad \tilde{a}\ll a_0, \end{aligned}$$

where $a_0 = \text {const}$ is the average background composition of the solution phase, $\tilde{a}(t)$ is the amplitude of the compositional fluctuation, and $K =2 \pi /\lambda$ is the wave number.

We now insert from Eq. (A-2) into Eq. (A-1). For the time derivative of *a* (left hand side of Eq. A-1) we have

A-3 $$\begin{aligned} \frac{\partial a}{\partial t} = \frac{\partial \tilde{a} }{\partial t} \cos Kx. \end{aligned}$$

Based on the notion that $a_0 = \text {const}$ and $\tilde{a}\ll a_0$ the right hand side of Eq. (A-1) is written as

A-4 $$\begin{aligned} \nabla \left[ \frac{D}{RT} a \left( 1-a \right) \nabla \left( \frac{ \partial g}{\partial a} - \kappa \nabla ^2 a \right) \right] = \frac{D}{RT} a_0 \left( 1-a_0 \right) \nabla ^2 \left( \frac{ \partial g}{\partial a} - \kappa \nabla ^2 a \right) . \end{aligned}$$

We expand $\partial g/\partial a$ as a Taylor series, which we truncate after the linear term

A-5 $$\begin{aligned} \frac{\partial g}{\partial a} \approx g_0'+g_0''(a-a_0)=g_0'+g_0''\tilde{a}\cos Kx, \end{aligned}$$

where

$$\begin{aligned} g_0'= \frac{\partial g}{\partial a}\bigg |_{a_0}=\text {const} \qquad \text {and}\qquad g_0''= \frac{\partial ^2 g}{\partial a^2}\bigg |_{a_0}=\text {const}. \end{aligned}$$

Inserting from Eq. (A-2) for *a* and from Eq. (A-5) for $\partial g/\partial a$ we obtain for the right hand side of Equation (A-1)

A-6 $$\begin{aligned} \frac{D}{RT}a_0( 1-a_0) \left[ -g_0''K^2 - \kappa K^4 \right] \tilde{a} \cos Kx . \end{aligned}$$

Combining Eq. (A-3) and (A-6), the growth rate of the amplitude of compositional fluctuations can be expressed as an explicit function of wave number

A-7 $$\begin{aligned} \frac{\partial \tilde{a}}{\partial t} = \frac{D}{RT}a_0 (1-a_0) \left[ -g_0''K^2 - \kappa K^4\right] \tilde{a}, \end{aligned}$$

or in terms of wavelength

A-8 $$\begin{aligned} \frac{\partial \tilde{a}}{\partial t} = \frac{D}{RT}a_0 (1-a_0) \left[ - \frac{4 \pi ^2g_0''}{ \lambda ^2}-\frac{16 \pi ^4\kappa }{ \lambda ^4}\right] \tilde{a}. \end{aligned}$$

The amplitude growth rate as a function of wavelength is shown in Fig. 6. Note that all coefficients in Eq. (A-8) are positive, except for $g_0''$, which is negative in the unstable region of the phase diagram. The amplitude of compositional fluctuations grows, when

$$\begin{aligned} - \frac{4 \pi ^2g_0''}{ \lambda ^2}-\frac{16 \pi ^4\kappa }{ \lambda ^4} > 0, \end{aligned}$$

which is the case for

$$\begin{aligned} \lambda> \lambda _{\text {min}} > 0, \end{aligned}$$

where $\lambda _{\text {min}}$ is obtained from

A-9 $$\begin{aligned} -\frac{4 \pi ^2g_0''}{ \lambda _{\text {min}}^2} - \frac{16 \pi ^4\kappa }{ \lambda _{\text {min}}^4} = 0, \end{aligned}$$

which yields

A-10 $$\begin{aligned} \lambda _{\text {min}} = \frac{2 \pi }{\sqrt{- g_0''/\kappa }}. \end{aligned}$$

Equation (A-10) defines the minimum length below which spinodal decomposition cannot occur.

The characteristic wavelength of the incipient compositional modulation is obtained from finding $\lambda _{\text {opt}}$, which maximizes $\frac{\partial \tilde{a}}{\partial t}$. To this end we set

A-11 $$\begin{aligned} \frac{d}{d \lambda } \left[ - \frac{4 \pi ^2g_0''}{ \lambda ^2} - \frac{16 \pi ^4\kappa }{ \lambda ^4} \right] = 0, \end{aligned}$$

and obtain

A-12 $$\begin{aligned} \lambda _{\text {opt}} = \frac{2 \pi }{\sqrt{-g_0''/(2 \kappa )}}=\sqrt{2}\,\lambda _{\text {min}}. \end{aligned}$$

Equation (A-12) defines the characteristic wavelength of the incipient chemical segregation during spinodal decomposition. Equation (A-12) can be re-arranged to express $\kappa$ as an explicit function of $\lambda _{\text {opt}}$ [see Eq. (4)].
